# Real world evaluation of a novel lateral flow assay (AlphaKit® QuickScreen) for the detection of alpha-1-antitrypsin deficiency

**DOI:** 10.1186/s12931-018-0826-8

**Published:** 2018-08-13

**Authors:** Timm Greulich, Francisco Rodríguez-Frias, Irene Belmonte, Andreas Klemmer, Claus F. Vogelmeier, Marc Miravitlles

**Affiliations:** 1 Department of Medicine, Pulmonary and Critical Care Medicine, University Medical Centre Giessen and Marburg, Philipps-University, Marburg, Germany; 2 German Centre for Lung Research (DZL), Marburg, Germany; 3 Liver Pathology Unit, Departments of Biochemistry and Microbiology, Hospital Universitari Vall d’Hebron, Universitat Autònoma de Barcelona (UAB), Barcelona, Spain; 4 CIBER de Enfermedades Hepáticas y Digestivas (CIBERehd), Instituto Nacional de Salud Carlos III, Madrid, Spain; 5 Pneumology Department, Hospital Universitari Vall d’Hebron, CIBER de Enfermedades Respiratorias (CIBERES), Barcelona, Spain; 6 Respiratory Medicine, University Hospital of Gießen and Marburg, 35043 Marburg, Germany

**Keywords:** Chronic obstructive pulmonary disease, COPD, Alpha-1-antitrypsin deficiency, Screening, Lateral flow assay

## Abstract

**Background:**

Alpha-1-Antitrypsin (AAT) deficiency (AATD) is a hereditary disorder that manifests primarily as pulmonary emphysema and liver cirrhosis. The clinically most relevant mutation causing AATD is a single nucleotide polymorphism Glu342Lys (Z-mutation). Despite the recommendation to test every COPD patient, the condition remains severely underdiagnosed with a delay of several years between first symptoms and diagnosis. The Grifols’ AlphaKit® QuickScreen is a novel qualitative point-of-care (POC) in vitro screening test developed for the detection of the Z AAT protein in capillary whole blood. The objective of this prospective, international, multi-center, diagnostic, interventional real-world study was to assess the performance of this device for the detection of AATD in test-naïve COPD patients.

**Methods:**

1044 test-naïve COPD patients were recruited from 9 centers in Spain and 10 centers in Germany, ranging from primary to tertiary care. To evaluate the performance of the test, sensitivity, specificity, positive predictive value (PPV), and negative predictive value (NPV) were calculated compared with the gold standard (genotyping).

**Results:**

Genotyping and phenotyping of all 1019 evaluable samples revealed 4.12% of patients as carriers of at least one Z-allele, while 0.29% carried the homozygous genotype Pi*ZZ. The evaluation of the test’s ability to detect the PiZ protein yielded the following results: specificity 97.8%, sensitivity 73.8%, negative predictive value 98.9%, and positive predictive value 58.5%. All false negatives (*n* = 11) were heterozygote Pi*MZ samples.

**Conclusions:**

The tested device can be used as an appropriate tool to exclude AATD in primary care and in the overall COPD population, except in patients with a high a-priori- probability of AATD.

## Background

Alpha-1-Antitrypsin (AAT) deficiency (AATD) is one of the most common hereditary disorders [1]. Clinical manifestations include pulmonary emphysema, liver cirrhosis, c-ANCA positive vasculitis and an inflammatory skin disease called panniculitis [2]. More than 150 genetic variants of coding gene SERPINA1 have been described [3] but the most common deficiency alleles are Pi*Z and Pi*S [4–7]. In Pi*ZZ, Pi*SZ and some other rare genotypes, the serum level of AAT is deficient [8–10].

According to the recent statement by the European Respiratory Society (ERS), diagnostic detection should target individuals with chronic pulmonary disease [11]. Similarly, in 1997 the WHO indicated that all patients with COPD should have their serum level of AAT tested [12]. However, despite these recommendations and the estimation that roughly 1% of subjects with COPD have AATD [2], the condition remains underdiagnosed [13, 14] with a typical delay of several years between first symptoms and diagnosis [15–17].

Although a number of detection initiatives and screening programs have been launched over the last several years [18, 19], including in the primary care setting [20], little progress has been made regarding the underdiagnosis and delayed diagnosis of AATD. Limited awareness and knowledge have been identified as the main barriers [21, 22], but a cumbersome testing algorithm may also have a negative impact on the motivation to test for AATD. Currently, AATD testing is based on the combination of different laboratory methods, such as measuring the AAT concentration in serum, followed by a phenotyping and genotyping analysis [23–26]. As this is a time- and cost-intensive approach, new tools allowing efficient and reliable detection of AATD at the primary care level are warranted.

The AlphaKit® QuickScreen (Diagnostic Grifols, Barcelona, Spain) is a novel qualitative in vitro screening test, with the characteristics of a point-of-care (POC) system, developed for protein Z detection in capillary whole blood [27]. The device is based on the lateral flow assay technology to deliver results within 15–30 min. It uses a mouse monoclonal antibody directed against the Z protein, which are dye labelled. In the event that a Z-variant is present in the blood, the labelled monoclonal antibody would bind to the Z protein in a “sandwich” position, drawing a red line in the reading panel (Z-AAT line). The Z-AAT signal will be positive for all Z allele carriers, regardless if the patient is heterozygous or homozygous for this gene.

In this international, multi-center, diagnostic, interventional real-world study, the performance of this novel device for the detection of AATD in test-naïve COPD patients was assessed.

## Methods

### Study design and objective

We performed a prospective, multi-center, international, diagnostic real-world study with the objective of assessing the performance of AlphaKit® QuickScreen, a novel vitro diagnostic screening device for AAT deficiency. The performance of the study was monitored by an external clinical research organization (ICON Clinical Research GmbH).

### Patients

AATD test-naïve COPD patients were recruited from 9 centers in Spain and 10 centers in Germany, ranging from primary to tertiary care. The samples were processed at the Hospital General Universitari Vall d’Hebron in Barcelona (Spain) and the University Medical Centre Giessen and Marburg (Germany). The inclusion criteria were: i) age ≥ 30; ii) diagnosed with COPD by post- bronchodilator FEV_1_/FVC < 0.7; iii) being in a stable medical state; and iv)understanding of the study and agreement to give written informed consent. The exclusion criterion was that the participant had not been subjected to any AAT deficiency test before.

Sites identified eligible patients based on their medical records as a pre-screening step. Screening and enrollment occurred on the same day, and only patients who signed the informed consent form were included in the study.

### Study device

The AlphaKit® QuickScreen is an immuno-chromatographic, in vitro diagnostic screening test for Z-AAT [27]. Each test requires 30 μL of capillary whole blood. The test is designated for use by qualified healthcare professionals only. The kit contains two variants of a monoclonal antibody against the Z-AAT protein. One is gold-labelled (detector) and the other one is immobilized on the surface on the test strip (capture). The sample (whole blood), together with the running buffer, releases the gold-labelled antibody (detector) out of its matrix. If the examined sample contains the Z-AAT protein, the antibody forms a complex with the analyte (gold-labelled anti-Z-AAT antibody - Z-AAT protein). This complex moves along the test strip by capillary action. At the “Z-AAT”-position, the “capture” anti-Z-AAT antibody is immobilized on the surface and binds the complex specifically via the Z-AAT protein. Depending on the concentration of Z-AAT within the sample, the intensity of the red/purple test line at the “Z-AAT”-position may vary. If the sample does not contain Z-AAT (e.g. Pi*MM), no complex can be formed and therefore no test line will appear. The excess gold-labelled antibodies bind at the control line (“Control”) indicating that the chromatography worked properly.

For Z-AAT detection, 30 μL of finger stick capillary blood were added into the sample reservoir of the screening device and, after waiting 1–2 min, 3 drops of running buffer were applied. Within 15–30 min, the results were read in the display.

### Testing device performance evaluation

Test performance was evaluated by analyzing the samples with the testing device and comparing the results with those obtained by genotyping.

**Fig. 1:**
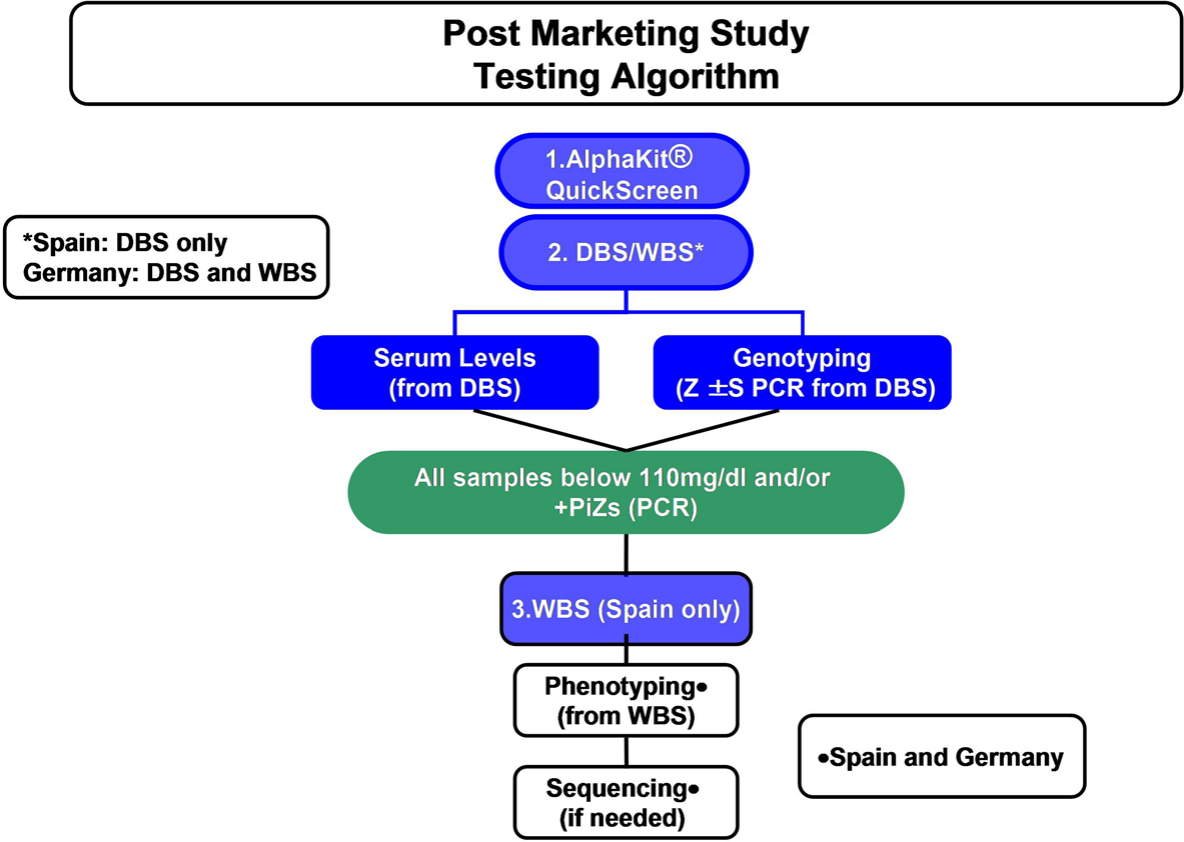
The testing algorithm is summarized. In both countries (Spain and Germany), the initial analysis consisted of the screening device (AlphaKit® QuickScreen) and the acquisition of blood on DBS (dried blood spot – AlphaKit). If the DBS-derived AAT serum level was ≥110 mg/dl and genotyping for the Z- and S-allele was negative, AATD was considered as excluded. All other samples underwent phenotyping to obtain a final result. DBS: dried blood spot; WBS: whole blood sample

Both positive and negative test results were confirmed independently and in a blinded manner by the central laboratory. For genotyping, the AlphaKit® (Diagnostic Grifols, Barcelona, Spain) test was used to collect finger stick capillary blood samples on the filter paper. After allowing the blood sample to dry for 1 h, the specimens were mailed to the testing laboratories in Marburg (Germany) or Barcelona (Spain) where genotyping for S and Z by means of the PCR technology and AAT level measurements were performed. The exact phenotype was determined in samples where the S or Z mutation was present (according to genotyping) or when the DBS-derived AAT level was < 110 mg/dl [23, 28]. The testing algorithm is summarized in Fig. 1.

### Data analysis

The sample size calculation was based on the assumptions that i) the percentage of COPD subjects with severe AATD Pi*ZZ was likely to be less than 1% in an unselected population; ii) the percentage of COPD subjects with heterozygous AATD Pi*MZ was assumed to be between 2 and 5%; iii) the percentage of COPD subjects with heterozygous AATD Pi*SZ was likely to be less than 1%, and iii) the total percentage of positive (any genotype combination with a Z-protein) with the tested screening device was expected to be between 2 and 5%. Therefore the number of subjects planned to be tested was 1000 (500 per country) with the expectation that 50 subjects would carry the Z-allele. The total number of enrolled subjects could be adjusted to exceed 1000, until 50 Z-positive samples were detected.

We performed descriptive statistics displaying continuous variables as mean ± standard deviation (SD), and categorical variables by number and percentage. To evaluate the performance of the test, sensitivity, specificity, positive predictive value (PPV), and negative predictive value (NPV) were calculated compared with the gold standard (genotyping). Discrepancies were shown as numbers of false positive (FP) and false negative (FN) results. Causes of discrepancies were analyzed.

From the study-derived sensitivity and specificity, a variety of predictive values were calculated to simulate the test-performance under a number of clinical scenarios with different prevalence (pre-test probability).

SPSS Version 22 (IBM, Ehningen, Germany), and GraphPad Prism Version 7 (GraphPad Software, Inc., La Jolla, USA) were used for calculations.

## Results

### Patients characteristics

One thousand and forty four test-naïve COPD patients that met the inclusion criteria were enrolled. Twenty-five of them were excluded: 20 due to missing results from the tested screening device or genotyping and 5 because the screening test was performed incorrectly.

The full analysis set consisted of 1019 patients, 549 from Germany and 470 from Spain. The mean age was 66.3 ± 9.7 years, the mean FEV_1_ was 53.0 ± 20.3%predicted, and the majority of the test population was male (69.3%). Baseline demographic and clinical characteristics of patients by country are shown in Table 1.

**Table 1:** Baseline characteristics of patients of the full analysis set

	Total population	Germany	Spain
N	1019	549	470
Age [years]	66.3 ± 9.7	65.4 ± 9.5	67.2 ± 9.7
% male	706 (69.3)	315 (57.4)	391 (83.2)
Duration of COPD [years]	6.7 ± 6.7	7.3 ± 6.8	5.9 ± 6.6
Emphysema	581 (57.0)	349 (63.6)	232 (49.4)
Chronic bronchitis	680 (66.7)	371 (67.6)	309 (65.7)
Bronchiectasis	75 (7.4)	25 (4.6)	50 (10.6)
Asthma	122 (12.0)	75 (13.7)	47 (10.0)
FEV_1_ [L]	1.47 ± 0.69	1.40 ± 0.72	1.55 ± 0.65
FEV_1_ [% predicted]	53.0 ± 20.7	50.2 ± 20.7	56.2 ± 20.3
FEV_1_/FVC [%]	53.1 ± 11.7	53.2 ± 12.2	53.0 ± 11.1
Exacerbations last year	564 (55.3)	273 (49.7)	291 (61.9)
Current smoker	267 (26.2)	135 (24.6)	132 (28.1)
Ex-smoker	721 (70.8)	395 (72.0)	326 (69.4)
Never smoker	31 (3.0)	19 (3.5)	12 (2.6)
Pack years	51.2 ± 36.4	45.4 ± 35.7	57.9 ± 36.1

Data displayed are the mean ± standard deviation for continuous variables, and n (%) for categorical variables

### Prevalence of pi*Z positive samples in the study population

Genotyping and phenotyping of all 1019 samples indicated that 4.12% of patients carried at least one Z-allele. The percentage of patients who carried the homozygous genotype Pi*ZZ was 0.29%. Results were similar in patients from Germany and Spain. Details for the S- and Z-allele by country are shown in Table 2.

**Table 2:** Genotypes/phenotypes according to the central laboratories test results. Values are reported as n (%)

Genotype	Total	Germany	Spain
Pi*ZZ	3 (0.29)	1 (0.18)	2 (0.42)
Pi*SZ	4 (0.39)	1 (0.18)	3 (0.63)
Pi*MZ	35 (3.43)	24 (4.37)	11 (2.34)
Any z	42 (4.12)	26 (4.74)	16 (3.40)

### Testing device performance

Results of test performance for detecting Z protein showed high specificity and NPV of the tested screening device when compared to genotyping (97.8 and 98.9%, respectively), while sensitivity and PPV resulted lower (73.8 and 58.5%, respectively). Detailed results of truly positive, truly negative, falsely positive and falsely negative samples are shown in Table 3. The analysis of false negatives showed that all 11 patients were heterozygote Pi*MZ.

**Table 3:** Performance for detecting Z protein of the tested device compared with the gold standard (genotyping)

		Genotyping		
		Positive	Negative	Total
Screening device	Positive	31	22	53
	Negative	11	955	966
	Total	42	977	1019

As the predictive values are based on the prevalence of the disease in the test population, we simulated the test-performance under a number of clinical scenarios (reported in the literature) with different prevalence (pre-test probability). The negative predictive value was high (> 98%) in unselected COPD populations (prevalence values taken from the literature) as well as in our study population, and decreased to lower numbers in a population with a very high pre-test probability (Table 4).

**Table 4:** Calculated predictive values in different clinical scenarios as reported in the literature

	Prevalence of Pi*Z positive carriers		NPV	PPV
European Population [4]	1:35	0.029	99.2	49.5
Primary Care in Spain [20]	1:31	0.032	99.1	52.0
Current Study	1:24	0.042	98.9	58.5
COPD II – IV in PFT [34]	1:22	0.046	98.7	61.2
Case finding in Germany [35]	1:30	0.303	89.6	93.4

*PFT* pulmonary function test, *NPV* negative predictive value, *PPV* positive predictive value

## Discussion

In this real-world study, we assessed the performance of a novel POC technology-based tool for the detection of AATD in test-naïve COPD patients. The tested device exhibited a high specificity and a moderate sensitivity.

More than 1000 samples were obtained from test-naïve COPD patients and compared to the gold standard, a combination of serum level, genotyping and – where applicable – phenotyping. The intended sample size was met and exceeded. The demographic and clinical profile of the studied population was the expected for the underlying pathology. Similarities between patients from both participating countries indicated that likely no selection bias took place.

The prevalence of samples carrying at least one Z-mutation was 4.1% in the overall test population (4.7% in Germany and 3.4% in Spain). The results of other targeted screening studies vary widely [29], but recent screening studies in Germany and Spain in test-naïve populations similar to our populations yielded remarkably similar results. In a study on 971 COPD patients in Spain, a total of 2.9% carriers of a Z allele was found [30], and in a population-based study, using health-care records in Catalonia, Barrecheguren et al. found that 5.3% of the test population exhibited an intermediated AAT serum level (50–100 mg/dl), most likely due to the presence of one Z-allele [31]. Similarly, Wencker et al. found a prevalence of 3.7% Pi*MZ in a German targeted test population [32].

In our study, the tested device performed with a specificity of 97.8% and sensitivity of 73.8%. In the prevalence observed, this corresponded to a positive predictive value of 58.5% and a negative predictive value of 98.9%. The positive predictive value of 58,5% clearly indicates that further testing (genotyping/phenotyping) is absolutely mandatory to confirm and clarify a positive test result. However, for a screening test, the most important indicator of performance is the negative predictive value, as it describes the level of certainty with which the disease can be excluded after having obtained a negative test result. Comparing the examined screening device with other tests that are widely used in clinical practice, it is interesting to note that for example procalcitonin (in combination with CRB-65) exhibited a negative predictive value of 98.8%, also [33]. Furthermore, because all false negatives were heterozygote Pi*MZ, all Pi*ZZ and Pi*SZ have been correctly identified.

The intermediate sensitivity of 73.8% may limit the applicability of the test in populations where the a-priory-probability is higher than in our current study population. As illustrated in Table 4, the test sufficiently rules out AATD in our test population as well as in other unselected COPD populations where the prevalence of the Z-allele may be around 5% [34], which would result in a negative predictive value of 98.5%. However, in clinical scenarios with an a-priory-probability of greater than 16.4% (examples could include COPD patients with an index case as a first degree relative or a young never-smoker with high-grade basal emphysema) the false negative rate would increase to values > 5%, probably judged as insufficient for use in that specific patient population. If a false negative rate of 10% would be judged as still acceptable, the prevalence of the population in which the test would be applicable, could be as high as 29.3%.

A further test-immanent limitation is that only deficient genotypes containing at least one Z allele can be detected [27]. In general, combinations of deficiency alleles without a Z-mutation such as Null/Null or S/Null are extremely rare [9, 35]. With the exception of countries like Italy where the prevalence of non-Z-mutations is considerably higher [36, 37], this may not limit the usability of the test.

A combination of different laboratory methods has been described as the optimal strategy for the diagnosis of AATD [23–26], and quantitative measurements of the AAT serum level are used widely as the initial screening test [38, 39], but the determination requires considerable time. Furthermore, as an acute phase reactant, levels of AAT may vary depending on clinical conditions. The tested screening device should not be seen as a diagnostic tool but could be used as a screening tool. Phenotyping and genotyping are necessary upon indication of a positive result. The test kit may have considerable potential to increase the overall AATD detection rate, simplifying the screening pathway. Early diagnosis is essential, as some individuals will qualify for augmentation therapy with AAT as well as benefit from lifestyle modifications.

Overall, these results demonstrate the tested device to be useful in primary care and in the overall COPD population, except in patients with a high a-priori-probability of AATD. In these patients, determination of the serum level and further diagnostic steps would be required.

## Conclusions

We performed an international, multi-center, diagnostic, interventional real-world study to assess the performance of AlphaKit® QuickScreen, a novel device for the screening of AATD in test-naïve COPD patients. With a negative predictive value of 98.9%, we conclude this novel screening device to be useful in primary care and in the overall COPD population, except in patients with a markedly increased a-priori-probability of AATD.

## References

[1] Stoller JK, Aboussouan LS (2005). Alpha1-antitrypsin deficiency. Lancet.

[2] Silverman EK, Sandhaus RA (2009). Clinical practice. Alpha1-antitrypsin deficiency. N Engl J Med.

[3] Demeo DL, Silverman EK (2004). Alpha1-antitrypsin deficiency. 2: genetic aspects of alpha(1)-antitrypsin deficiency: phenotypes and genetic modifiers of emphysema risk. Thorax.

[4] Blanco I, de Serres FJ, Fernandez-Bustillo E, Lara B, Miravitlles M (2006). Estimated numbers and prevalence of PI*S and PI*Z alleles of alpha1-antitrypsin deficiency in European countries. Eur Respir J.

[5] Greene CM, Miller SD, Carroll T, McLean C, O'Mahony M, Lawless MW, O'Neill SJ, Taggart CC, McElvaney NG (2008). Alpha-1 antitrypsin deficiency: a conformational disease associated with lung and liver manifestations. J Inherit Metab Dis.

[6] Lomas DA, Evans DL, Finch JT, Carrell RW (1992). The mechanism of Z alpha 1-antitrypsin accumulation in the liver. Nature.

[7] Fregonese L, Stolk J (2008). Hereditary alpha-1-antitrypsin deficiency and its clinical consequences. Orphanet J Rare Dis.

[8] de Serres FJ, Blanco I, Fernandez-Bustillo E (2006). Estimating the risk for alpha-1 antitrypsin deficiency among COPD patients: evidence supporting targeted screening. COPD.

[9] Blanco I, Bueno P, Diego I, Perez-Holanda S, Casas-Maldonado F, Esquinas C, Miravitlles M (2017). Alpha-1 antitrypsin pi*Z gene frequency and pi*ZZ genotype numbers worldwide: an update. Int J Chron Obstruct Pulmon Dis.

[10] Blanco I, Bueno P, Diego I, Perez-Holanda S, Lara B, Casas-Maldonado F, Esquinas C, Miravitlles M (2017). Alpha-1 antitrypsin pi*SZ genotype: estimated prevalence and number of SZ subjects worldwide. Int J Chron Obstruct Pulmon Dis.

[11] Miravitlles M, Dirksen A, Ferrarotti I, Koblizek V, Lange P, Mahadeva R, McElvaney NG, Parr D, Piitulainen E, Roche N, et al. European Respiratory Society statement: diagnosis and treatment of pulmonary disease in alpha1-antitrypsin deficiency. Eur Respir J. 2017;50 10.1183/13993003.00610-2017 29191952

[12] WHO (1997). Alpha 1-antitrypsin deficiency: memorandum from a WHO meeting. Bull World Health Organ.

[13] de Serres FJ, Blanco I (2012). Prevalence of alpha1-antitrypsin deficiency alleles PI*S and PI*Z worldwide and effective screening for each of the five phenotypic classes PI*MS, PI*MZ, PI*SS, PI*SZ, and PI*ZZ: a comprehensive review. Ther Adv Respir Dis.

[14] Stoller JK, Brantly M (2013). The challenge of detecting alpha-1 antitrypsin deficiency. COPD.

[15] Campos MA, Wanner A, Zhang G, Sandhaus RA (2005). Trends in the diagnosis of symptomatic patients with alpha1-antitrypsin deficiency between 1968 and 2003. Chest.

[16] Kohnlein T, Janciauskiene S, Welte T (2010). Diagnostic delay and clinical modifiers in alpha-1 antitrypsin deficiency. Ther Adv Respir Dis.

[17] Stoller JK, Sandhaus RA, Turino G, Dickson R, Rodgers K, Strange C (2005). Delay in diagnosis of alpha1-antitrypsin deficiency: a continuing problem. Chest.

[18] Chorostowska-Wynimko J (2015). Targeted screening programmes in COPD: how to identify individuals with alpha1-antitrypsin deficiency. Eur Respir Rev.

[19] Corda L, Bertella E, Pini L, Pezzini A, Medicina D, Boni E, Guerini M, Trivella S, Grassi V, Tantucci C (2006). Diagnostic flow chart for targeted detection of alpha1-antitrypsin deficiency. Respir Med.

[20] Molina J, Flor X, Garcia R, Timiraos R, Tirado-Conde G, Miravitlles M (2011). The IDDEA project: a strategy for the detection of alpha-1 antitrypsin deficiency in COPD patients in the primary care setting. Ther Adv Respir Dis.

[21] Esquinas C, Barrecheguren M, Sucena M, Rodriguez R, Fernandez S, Miravitlles M. Practice and knowledge about diagnosis and treatment of alpha-1 antitrypsin deficiency in Spain and Portugal. BMC Pulm Med. 2016;16

[22] Greulich T, Ottaviani S, Bals R, Lepper PM, Vogelmeier C, Luisetti M, Ferrarotti I (2013). Alpha1-antitrypsin deficiency - diagnostic testing and disease awareness in Germany and Italy. Respir Med.

[23] Bals R, Koczulla R, Kotke V, Andress J, Blackert K, Vogelmeier C (2007). Identification of individuals with alpha-1-antitrypsin deficiency by a targeted screening program. Respir Med.

[24] Ferrarotti I, Scabini R, Campo I, Ottaviani S, Zorzetto M, Gorrini M, Luisetti M (2007). Laboratory diagnosis of alpha1-antitrypsin deficiency. Transl Res.

[25] Snyder MR, Katzmann JA, Butz ML, Wiley C, Yang P, Dawson DB, Halling KC, Highsmith WE, Thibodeau SN (2006). Diagnosis of alpha-1-antitrypsin deficiency: an algorithm of quantification, genotyping, and phenotyping. Clin Chem.

[26] Miravitlles M, Herr C, Ferrarotti I, Jardi R, Rodriguez-Frias F, Luisetti M, Bals R (2010). Laboratory testing of individuals with severe alpha1-antitrypsin deficiency in three European centres. Eur Respir J.

[27] Vogelmeier C, Soriano J, Janciauskiene S, Crystal RG, Ferrarotti I, Carroll T. Alpha-1-antitrypsin deficiency: honouring the past and embracing the future. Grifols' symposium report. Expert Rev Respir Med. 2013;(Special Edition):1–10.

[28] Rodriguez F, Jardi R, Costa X, Cotrina M, Galimany R, Vidal R, Miravitlles M (2002). Rapid screening for alpha1-antitrypsin deficiency in patients with chronic obstructive pulmonary disease using dried blood specimens. Am J Respir Crit Care Med.

[29] Greulich T, Vogelmeier CF (2016). Alpha-1-antitrypsin deficiency: increasing awareness and improving diagnosis. Ther Adv Respir Dis.

[30] de la Roza C, Rodriguez-Frias F, Lara B, Vidal R, Jardi R, Miravitlles M (2005). Results of a case-detection programme for alpha1-antitrypsin deficiency in COPD patients. Eur Respir J.

[31] Barrecheguren M, Monteagudo M, Simonet P, Llor C, Rodriguez E, Ferrer J, Esquinas C, Miravitlles M (2016). Diagnosis of alpha-1 antitrypsin deficiency: a population-based study. Int J Chron Obstruct Pulmon Dis.

[32] Wencker M, Marx A, Konietzko N, Schaefer B, Campbell EJ (2002). Screening for alpha1-pi deficiency in patients with lung diseases. Eur Respir J.

[33] Kruger S, Ewig S, Marre R, Papassotiriou J, Richter K, von Baum H, Suttorp N, Welte T, Group CS (2008). Procalcitonin predicts patients at low risk of death from community-acquired pneumonia across all CRB-65 classes. Eur Respir J.

[34] Rahaghi FF, Sandhaus RA, Brantly ML, Rouhani F, Campos MA, Strange C, Hogarth DK, Eden E, Stocks JM, Krowka MJ, Stoller JK (2012). The prevalence of alpha-1 antitrypsin deficiency among patients found to have airflow obstruction. COPD.

[35] Greulich T, Nell C, Herr C, Vogelmeier C, Kotke V, Wiedmann S, Wencker M, Bals R, Koczulla AR (2016). Results from a large targeted screening program for alpha-1-antitrypsin deficiency: 2003 - 2015. Orphanet J Rare Dis.

[36] Ferrarotti I, Baccheschi J, Zorzetto M, Tinelli C, Corda L, Balbi B, Campo I, Pozzi E, Faa G, Coni P (2005). Prevalence and phenotype of subjects carrying rare variants in the Italian registry for alpha1-antitrypsin deficiency. J Med Genet.

[37] Ferrarotti I, Carroll TP, Ottaviani S, Fra AM, Brien O, Molloy K, Corda L, Medicina D, Curran DR, McElvaney NG, Luisetti M (2014). Identification and characterisation of eight novel SERPINA1 null mutations. Orphanet J Rare Dis.

[38] Steiner SJ, Gupta SK, Croffie JM, Fitzgerald JF (2003). Serum levels of alpha1-antitrypsin predict phenotypic expression of the alpha1-antitrypsin gene. Dig Dis Sci.

[39] Bornhorst JA, Greene DN, Ashwood ER, Grenache DG (2013). alpha1-antitrypsin phenotypes and associated serum protein concentrations in a large clinical population. Chest.

